# Status of serum magnesium in Egyptian children with type 1 diabetes and its correlation to glycemic control and lipid profile

**DOI:** 10.1097/MD.0000000000005166

**Published:** 2016-11-28

**Authors:** Doaa Shahbah, Amr Abo El Naga, Tamer Hassan, Marwa Zakaria, Mohamed Beshir, Salah Al Morshedy, Mohamed Abdalhady, Ezzat Kamel, Doaa Abdel Rahman, Lamiaa Kamel, May Abdelkader

**Affiliations:** a Department of Pediatrics; b Department of Clinical Pathology, Zagazig University, Egypt.

**Keywords:** children, diabetes, lipid, magnesium

## Abstract

Diabetes mellitus has been suggested to be the most common metabolic disorder associated with magnesium deficiency, having 25% to 39% prevalence. This deficit could be associated with the development of late diabetic complications, especially macroangiopathy.

We aimed to evaluate the status of serum Mg in children with type 1 diabetes and assess its relation to glycemic control and lipid profile.

We included 71 Egyptian children with type 1diabetes having their follow-up at Pediatric Endocrinology outpatient clinic, Zagazig University Hospital and 71 age- and sex-matched control. We measured Serum magnesium, HbA1c, and lipid profile in all study subjects.

Diabetic children had significantly lower serum magnesium level compared to control children (1.83 ± .27 mg/dL in diabetic children versus 2.00 ± .16 mg/dL in control children). Taking cut-off level of serum magnesium <1.7 mg/dL for definition of hypomagnesemia, hypomagnesemia was detected in 28.2% of diabetic children compared to 9.9% of control children. In diabetic patients, there was statistically significant difference in HbA1c between hypomagnesemic and normomagnesemic group being higher in the low magnesium group, as it is mean ± SD was 11.93 ± 3.17 mg/dL in group I versus 8.92 ± 0.93 mg/dL in the normomagnesemic group. Serum magnesium was found to be positively correlated with HDL (*P* < 0.001), and negatively correlated with age, HbA1c, triglycerides, total cholesterol, LDL, and duration of diabetes (*P* < 0.001).

We concluded that total serum magnesium was frequently low in Egyptian children with type 1 diabetes and it is correlated with HbA1c and with lipid profile. Hypomagnesemia was more evident in patients with poor diabetic control and those with higher atherogenic lipid parameters. We suggest that low serum magnesium may be included in pathogenesis of poor glycemic control and abnormal lipid profile in children with type 1 diabetes. We need to perform further studies on giving magnesium supplements in diabetic children with hypomagnesemia to observe the effect of correction of serum magnesium on glycemic control, lipid profile, and the risk of diabetic complications.

## 1 Introduction

Type 1 diabetes is a disorder that arises following the autoimmune destruction of insulin-producing pancreatic β cells^[1,2]^ The disease is most often diagnosed in children and adolescents, usually presenting with a classic trio of symptoms (i.e., polydipsia, polyphagia, and polyuria) alongside of overt hyperglycemia, positing the immediate need for exogenous insulin replacement—a medicinal introduction to the disorder whose therapeutic practice lasts a lifetime.^[3]^

Type 2 diabetes mellitus is emerging as a new clinical problem within pediatric practice. Recent reports indicate an increasing prevalence of type 2 diabetes mellitus in children and adolescents around the world in all ethnicities, even if the prevalence of obesity is not increasing any more. Type 2 diabetes mellitus is a complex metabolic disorder of heterogeneous etiology with social, behavioral, and environmental risk factors unmasking the effects of genetic susceptibility.^[4]^ There is a strong hereditary (likely multigenic) component to the disease, with the role of genetic determinants illustrated when differences in the prevalence of type 2 diabetes mellitus in various racial groups are considered.^[5]^ Furthermore, the recent increases observed in diabetes mellitus prevalence are too quickly to be the result of increased gene frequency and altered gene pool, emphasizing the importance of environmental factors. Treatment of choice is lifestyle intervention followed by pharmacological treatment (e.g., metformin). New drugs such as dipeptidyl peptidase inhibitors or glucagon like peptide 1 mimetics are in the pipeline for treatment of youth with type 2 diabetes mellitus.^[6]^

Increasing attention has been given to the role of certain elements in the pathogenesis of diabetes mellitus and in the progression of its complications.^[7]^

Diabetes mellitus (DM) is associated with alteration of the metabolism of micronutrients with magnesium (Mg) being the most studied in this regard. Mg plays an important role in carbohydrate metabolism; it may influence the release and activation of insulin, the hormone that helps to control blood glucose levels.^[8]^

Magnesium is the fourth most abundant cation in the body and its vast majority is stored intracellularly. The major organs involved in magnesium homeostasis are the gut, bone, and kidney, but the regulators affecting these organs at the cellular level are not yet fully understood.^[9]^

The role of magnesium in the body is widespread. It is an essential cofactor of more than 300 enzymes including those important in glycolysis, transcellular ion transport, neuromuscular transmission, synthesis of carbohydrates, proteins, lipid and nucleic acids, and release of end response to certain hormones.^[10]^ Diabetes mellitus has been suggested to be the most common metabolic disorder associated with magnesium deficiency, having 25 to 39% prevalence.^[11]^

Numerous causes for low magnesium levels in diabetics can be listed including diets low in magnesium, osmotic diuresis that leads to high renal excretion of magnesium, insensitivity to insulin that affects intracellular magnesium transport and causes increased loss of extracellular magnesium, usage of loop and thiazide diuretics that promote magnesium wasting, diabetic autonomic neuropathies, and reduced tubular reabsorption due to insulin resistance. Additionally, continuous magnesium deficiency correlates to higher levels of TNFα, which may also contribute to post-receptor insulin resistance.^[12]^

Such Mg deficits have been linked to the development of atherosclerosis,^[13,14]^ and in patients with coronary atherosclerosis, a Mg deficit has been related to an atherogenic lipid profile.^[15]^ Therefore, Mg deficit in patients with type 1diabetes could be associated with the development of late diabetic complications, especially macroangiopathy.^[16]^

Several studies were focused on evaluating Mg status in patients with type 2 diabetes and on role of Mg supplementation in prevention of diabetic complications and optimization of diabetic control. However; few studies have been concerned with this issue in children with type 1diabetes with opposing results. We aimed to evaluate the status of serum Mg in Egyptian children with type 1 diabetes and assess its relation to glycemic control and lipid profile. We found in our study a higher percentage of hypomagnesaemia in diabetic children relative to controls. Also we observed a negative correlation between serum magnesium and each of HBA1c and serum triglycerides, total cholesterol, LDL as well as duration of diabetes. However, there was a positive correlation between serum magnesium and HDL level. These results draw our attention to importance of monitoring serum magnesium level in children with type 1 diabetes, monitoring patients for possible complications associated with hypomagnesaemia. Whether hypomagnesaemia detected is a cause or consequence of bad diabetic control is not known.

## 2 Methods

A gender and age matched case control study was conducted at Pediatric Endocrinology Outpatient Clinic, Zagazig University, Egypt, during the period from February 2014 to December 2014.

### 2.1 Inclusion criteria

Subjects who met the following criteria were consecutively enrolled during their follow up at Pediatric Endocrinology Outpatient Clinic, Zagazig University

1. Type 1 diabetes
2. Age between 1 and 18 years.
3. Both sexes

### 2.2 Exclusion criteria

1. Children who have renal disease detected by serum urea and creatinine test
2. Diuretics usage in the last 2 weeks.
3. Children with persistent diarrhea and vomiting.

### 2.3 Study groups

1. Patient group: 71 children with type I diabetes.
2. Control group: 71 age- and sex-matched healthy subjects recruited form community.

### 2.4 Methods

All subjects underwent the following:

1. Thorough history taking and complete physical examination.
2. Routine investigations according to our local standards every 3 months, for example, complete blood count, renal function tests, random blood glucose, glycosylated hemoglobin, and so on.
3. Special investigations:
  a. Serum magnesium level by Integra 400 Plus (Roche, Germany).
  b. Lipid profile by Integra 400 Plus (Roche, Germany).

### 2.5 Ethics

The protocol developed is according to Declaration of Helsinki 1964, as revised in 2000, and was approved by the institutional review board at our faculty. Informed consent was obtained from all patients’ guardians prior to participation.

### 2.6 Statistical analysis

All data were collected, tabulated and statistically analyzed using SPSS 15.0 for windows (SPSS Inc., Chicago, IL). Continuous Quantitative variables, for example, age were expressed as the mean ± SD & median (range), and Categorical Qualitative variables were expressed as absolute frequencies “number” & relative frequencies (percentage).

All values are expressed as median and range or mean ± SD. Differences between groups were assessed by paired Student's *t* test or the Mann–Whitney *U* (MW) test. Correlation between variables was assessed using Spearman rank correlation coefficient. A *P* value of <0.05 was considered significant.

## 3 Results

The study included 71 patients with type 1 diabetes (32 males and 39 females); their mean age was 9.68 ± 3.99 years and that of 71 control children (43 males and 28 females) was 9.48 ± 3.23 years. Diabetic children had significantly lower serum magnesium level compared to control children (1.83 ± .27 mg/dL in diabetic children versus 2.00 ± .16 mg/dL in control children). Taking cut off level of serum magnesium <1.7 mg/dL for definition of hypomagnesemia,^[17]^ hypomagnesemia was detected in 28.2% of diabetic children compared to 9.9% of control children (Table 1). There were significant differences in lipid profile parameters between diabetic and control group with lower mean value of HDL in the diabetic group versus control group with *P* < 0.01 and higher mean values of other lipid parameters in diabetic group versus control group with *P* < 0.05 (Table 2). In diabetic children, serum magnesium was found to be positively correlated with HDL, MCV, and platelet count (*P* < 0.001), and negatively correlated with age, HbA1c, triglycerides, total cholesterol, LDL, and duration of diabetes (*P* < 0.001) (Table 3).

**Table 1:**
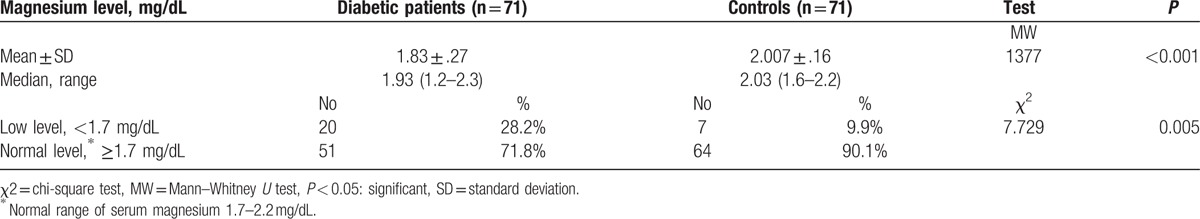
Comparison between diabetic patients and controls as regard serum magnesium level.

**Table 2:**
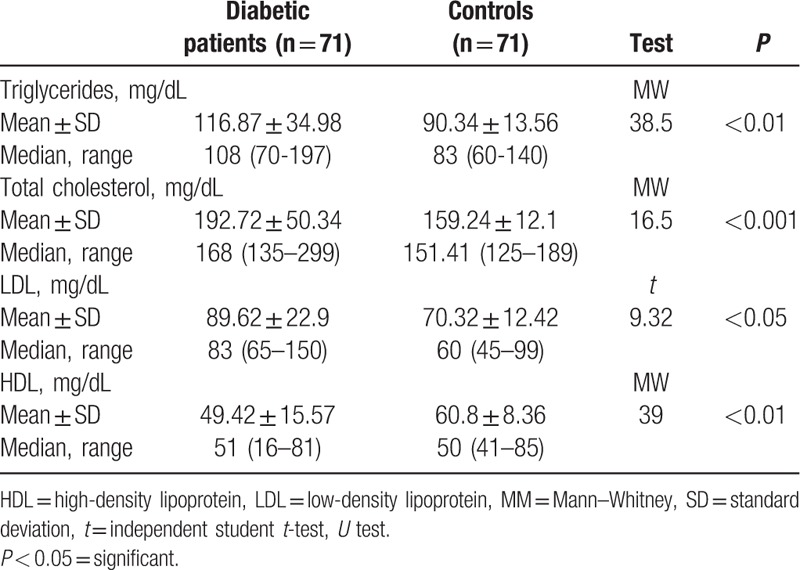
Lipid profile of diabetic patients and controls.

**Table 3:**
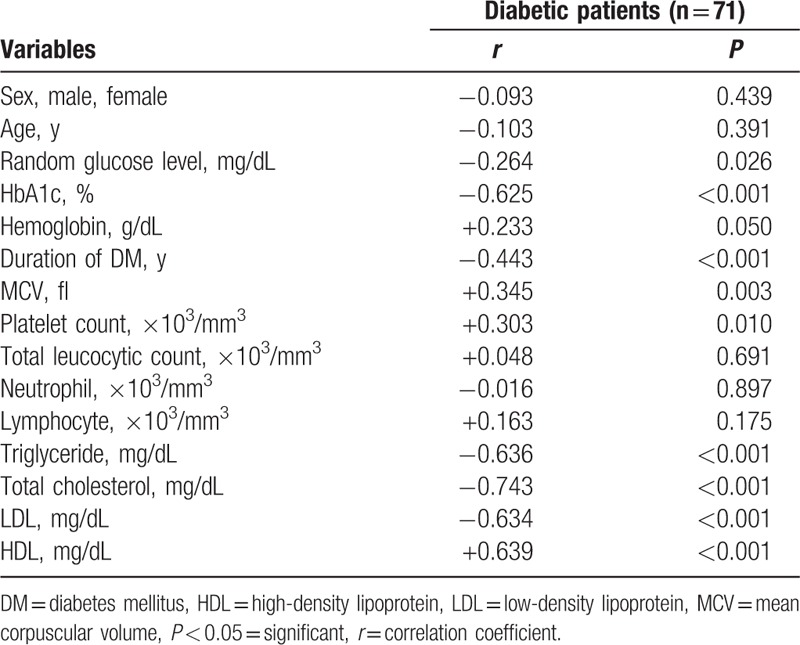
Correlations between serum magnesium level and some study parameters in diabetic patients.

There was statistically significant difference in duration of diabetes between the hypomagnesemic and normomagnesemic diabetic groups, as mean ± SD of diabetes duration was 4.8 ± 2.39 years in the hypomagnesemic group versus 1.94 ± 1.45 years in normomagnesemic group with *P* < 0.001.

There was statistically significant difference in HbA1c between hypomagnesemic group and normomagnesemic diabetic group with higher value of HbA1c in the low magnesium group, as mean ± SD was 11.93 ± 3.17% in group I versus 8.92 ± .93% in the normomagnesemic group.

The age was statistically different between normomagnesemic and hypomagnesemic diabetic children, being higher in hypomagnesemic one with mean ± SD was 10.11 ± 0.87 years versus 9.03 ± 3.51 years in the normomagnesemic group.

**Figure 1:**
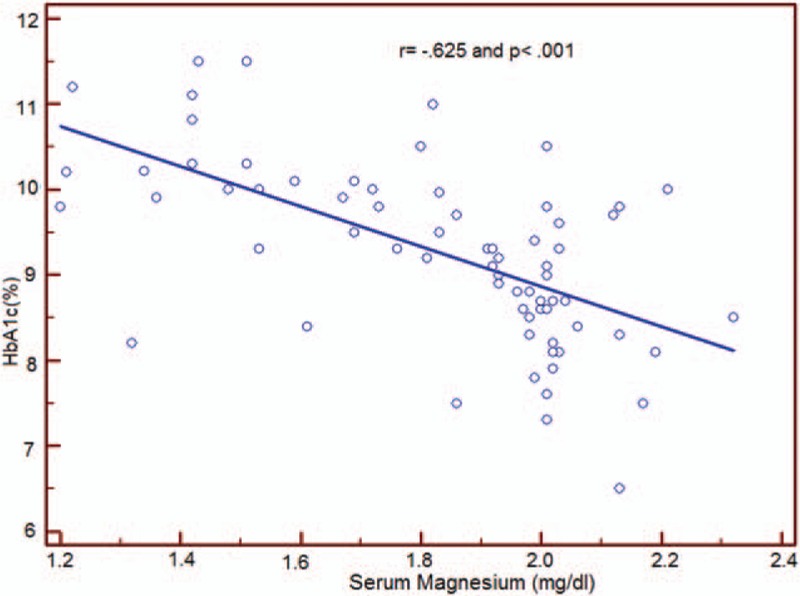
This figure shows that there is significantly negative correlation between serum magnesium and HbA1c.

According to Galli and Maggana^[17]^ we divided our study group into 4 quartiles based on serum magnesium level, quartile (Q)1, serum magnesium <1.67 mg/dL; Q2, serum magnesium 1.67–1.93 mg/dL; Q3, serum magnesium 1.93–2.02; Q4, serum magnesium >2.02. We found that Q1 that has the lowest value of serum Mg (<1.67 mg/dL), has the longest duration of diabetes (mean = 4.68) and the highest HbA1c (mean = 10.15) (Table 4). A negative correlation was found between serum magnesium level and HbA1c (*r* = −0.625, *P* < 0.001) (Fig. 1). Q1 also was associated with lowest serum HDL level and highest value of other serum lipid parameters (*P* < 0.001) (Table 5).

**Table 4:**
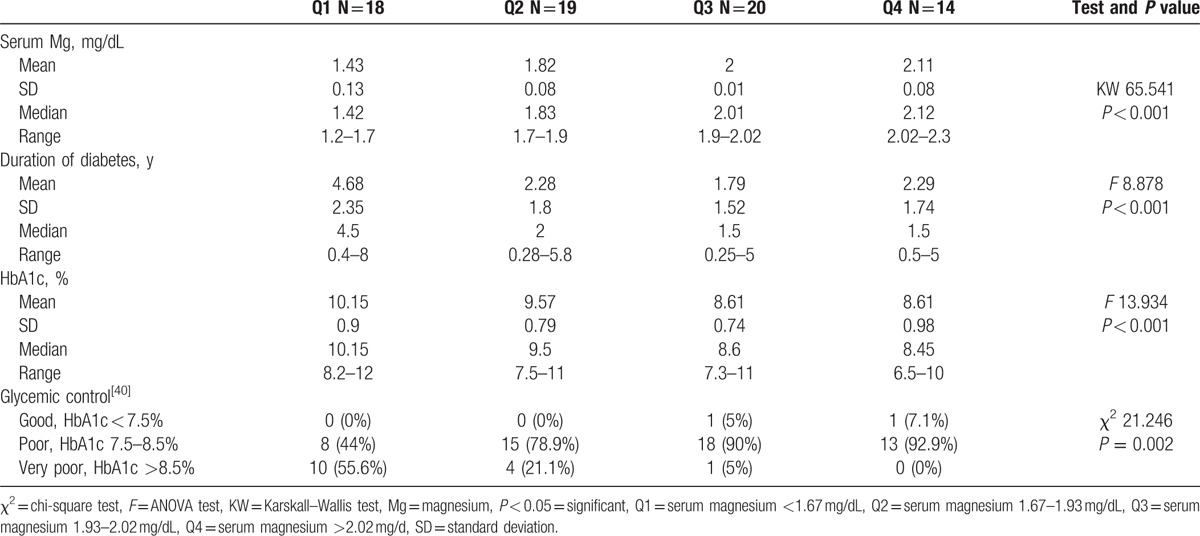
Comparison of serum magnesium concentration quartiles as regard clinical and laboratory findings.

**Table 5:**
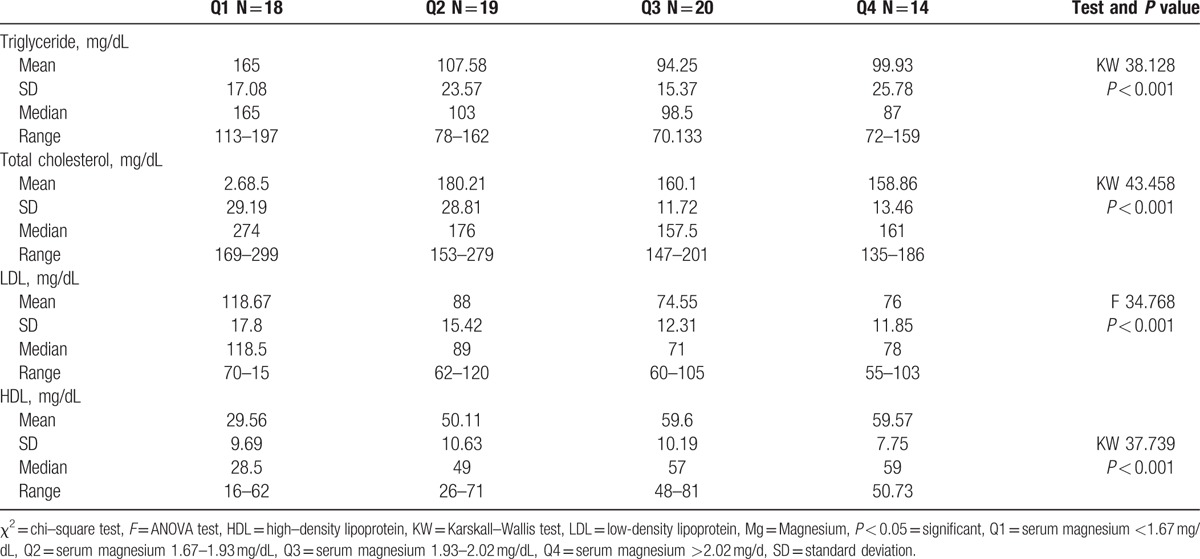
Comparison of serum magnesium concentration quartiles as regard the lipid profile.

**Figure 2:**
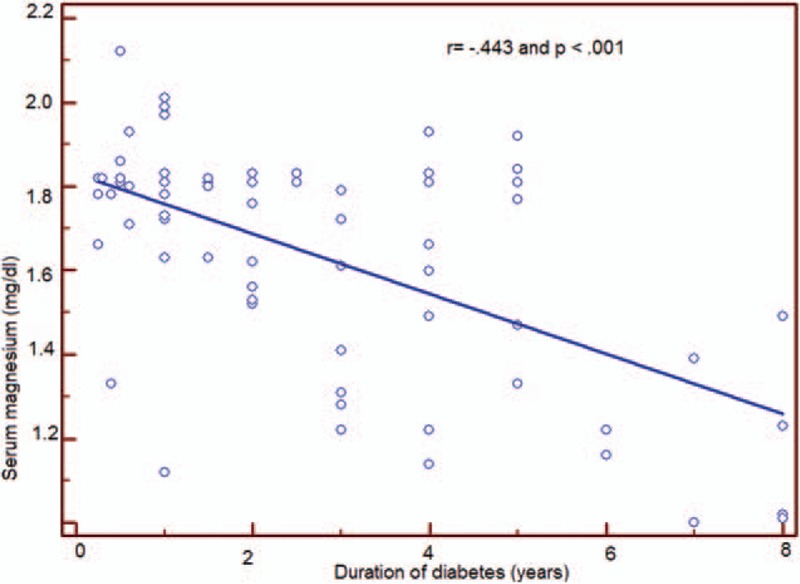
This figure shows that there is significantly negative correlation between serum magnesium and duration of diabetes.

There are many factors affecting serum magnesium level in diabetic cases, including age, sex, and duration of diabetes (Table 6). The most contributing factor to hypomagnesemia was duration of diabetes which exhibited statistically high significant difference (*P* < 0.001) (Fig. 2).

**Table 6:**
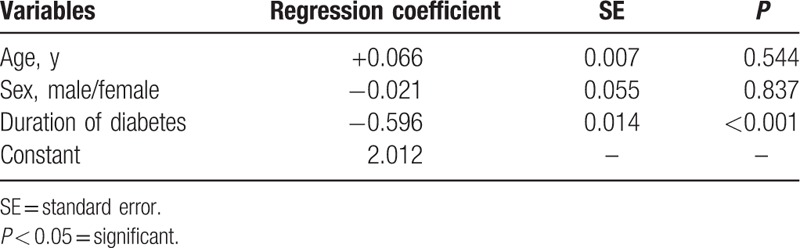
Multivariate linear regression of potential predictors of serum magnesium level in diabetic patients.

There are many factors affecting HbA1c level in diabetic cases, including age, sex, duration of diabetes, and serum magnesium level. The most contributing factor was serum magnesium which exhibited statistically high significant difference (*P* < 0.001) (Table 7).

**Table 7:**
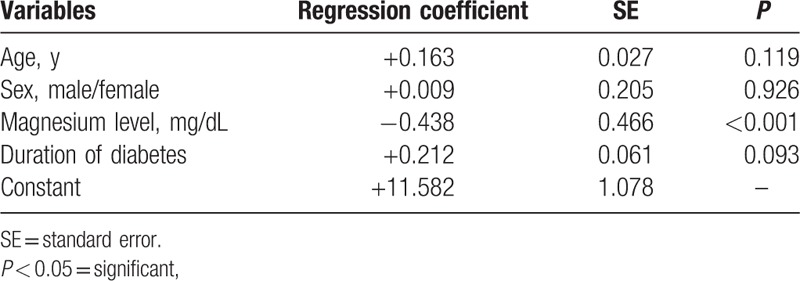
Multivariate linear regression of potential predictors of HbA1c level in diabetic patients.

## 4 Discussion

Type I diabetes mellitus is the most life-threatening endocrine disorder of children and its incidence appears to be increasing.^[18]^

Diabetes mellitus is the most chronic disease studied with respect to serum Mg level. Magnesium plays a significant role in glucose and insulin metabolism, mainly through its impact on tyrosine kinase enzyme, magnesium may directly affect Glucose Transporter protein activity 4 and regulate glucose translocation into the cell.^[19]^

The serum Mg level has been assessed in type 2 DM in different studies. However, there are few studies, concerned with serum magnesium and its relation to diabetic control in type I diabetes. Among these studies, there was a great controversy in results regarding frequency of hypomagnesemia and correlation between magnesium level and HbA1c.

### 4.1 Percentage of hypomagnesaemia in study groups

In our study, we found statistically significant difference in percentage of hypomagnesemia between diabetic patients and control group being higher in diabetic group, as percentage was 28.2% in the diabetic group versus 9.9% in the control group with lower level of serum magnesium in patients (1.83 mg/dL) versus (2.00 mg/dL) in control children. These results are in concordance with Jiancheng et al^[20]^ on 25 children with type I diabetes from Northeast area of China that revealed a lower serum magnesium level in type I diabetic children compared to control subjects. Lin et al,^[21]^ Salmonowicz et al,^[22]^ and Bjelakovic et al^[23]^ showed also similar results in type 1 diabetic children and adolescents compared to healthy controls. Indeed Fort and Lifshitz^[24]^ and Tuvemo et al^[25]^ have found a lower serum total magnesium in type 1 diabetic children especially those with poor glycemic control when compared to their healthy age- and sex-matched control Also, our results are concordant with Seyoum et al^[26]^ that found a higher percentage of hypomagnesemia (65%) among adults with type I and type II diabetes compared to control group and that is considered higher than that the percentage in our study. This may be due to the difference in the age of study population between it and ours. However, Hussmann et al^[27]^ shows a reduction in ionized serum magnesium with preserved total serum magnesium in children with type 1 diabetes relative to their healthy control.

Inconsistent with our results, Matthiesen et al^[28]^ failed to show any statistically significant difference in serum ionized Mg between Danish children with type I diabetes mellitus and control group, and also opposite to our result, Mikhail and Ehsanipoor^[29]^ showed that serum ionized Mg was significantly higher in type 2 diabetic patients than controls. This difference may be related to different studies populations between us and them. Moreover, poorer glycemic control in Egyptian children when compared to Danish children as represented by HBA1c. Also, in our study, we measured the total serum magnesium and not the ionized one unlike the previous 2 studies. Roffi et al^[30]^ showed similar concentration of serum magnesium in type 1 diabetic children and their control but their *study* was focused mostly on newly diagnosed type 1 diabetes patients only. This is considered supportive to our results as we found that hypomagnesemia is seen mainly in T1DM children with longer duration of diabetes.

### 4.2 Correlation of serum magnesium to glycemic control

By dividing study subjects to 4 quartiles based on the serum magnesium level, we found that Q 1 with the lowest serum magnesium level was associated with longer duration of diabetes, higher HbA1c, and poor glycemic control versus other quartiles with higher serum magnesium. We observed also a negative correlation between serum magnesium level and HbA1c (*r* = –0.625, *P* < 0.001). This agreed with Galli and Maggana ^[17]^ on Athens type I diabetic children that showed a lower Mg level in patients with poor glycemic control with high HbA1c. Ramadass et al^[31]^, Sinha and Sen^[32]^ showed similar results in adult patients with type II diabetes. Also Mikhail and Ehsanipoor^[29]^ observed an inverse correlation between total Mg and HbA1c.

Inconsistent with our result, Lin et al^[21]^ and Salmonowicz et al^[22]^ did not show any correlation between serum magnesium level and HbA1c in type 1 diabetic children and adolescents. Also, Matthiesen et al^[28]^ did not observe any relationship between HbA1c and ionized magnesium and Wegner et al^[33]^ failed to show any relation between serum magnesium and diabetic controls in the form of Fasting Blood Glucose. Again this difference between these studies and ours could be attributed to the difference in study populations, degree of diabetic control among them, also to the different methods of evaluating serum magnesium and glycemic control.

### 4.3 Correlation of serum magnesium with lipid profile

Our study showed that there was a significant difference between diabetic and control subjects as regard to lipid parameters with higher values of triglycerides (*P* < 0.01), total cholesterol (*P* < 0.001), and LDL (*P* < 0.05), in the diabetic group than control group. However, high-density lipoprotein was lower in the diabetic group than in the control group (*P* < 0.01).

This is in agreement with Wang et al^[34]^ and Mishra et al^[35]^ on adult population type II diabetes that showed a significant difference in lipid parameters between control and diabetic group with lower HDL and higher other lipid parameters in the diabetic group. Rasheed et al^[8]^ study on type II diabetic patients also detected a higher level of TG and lower value of HDL in type 2 diabetic group relative to control group (*P* = 0.03)

This lipid disturbance (higher triglycerides, total cholesterol and LDL with lower HDL) is most pronounced in diabetic children with hypomagnesemia compared to those with normal serum magnesium. A negative correlation is noted between serum Mg and triglycerides (*r* = −0.636, *P* < 0.001), total cholesterol (*r* = −0.743, *P* < 0.001) and LDL (*r* = −0.634, *P* < 0.001). However, there was a positive correlation between serum Mg and HDL (*r* = 0.639, *P* < 0.001).

These results are in concordance with Mishra et al^[35]^ study on adult type II diabetic patients that revealed a negative correlation between serum Mg and triglycerides (*r* = −0.519, *P* < 0.01) and a positive correlation with HDL (*r* = 0.741), but did not show any significant correlation between total cholesterol or LDL with serum Mg. Moreover, Srinivasau et al^[36]^ revealed a negative correlation between serum Mg and triglycerides (*P* < 0.05) especially in poorly controlled diabetics (*r* = −0.632) and Rasheed et al^[8]^ revealed a positive correlation of serum Mg with HDL (*r* = 0.34, *P* < 0.01), but there was a non-significant correlation with other lipid parameters.

Guerrero-Romero and Rodríguez-Morán^[37]^ showed that in patients with T2DM, hypomagnesemia is linked with low levels of HDL, irrespective of serum glucose level.

Our results are inconsistent with Wegner et al^[33]^ on type I diabetic children that found that there was no significant difference in lipid parameters between patients with normal and those with low serum magnesium level. Also, Jiancheng et al^[20]^ failed to show any association between serum magnesium and lipid profile in diabetic patients with or without complications.

In our study, we detected a negative correlation between serum magnesium level in diabetic children with duration of diabetes (*r* = −0.443, *P* < 0.001). This is consistent with a study on adults with T2DM by Mishra et al^[35]^ that showed a negative correlation between the serum magnesium level and duration of diabetes (*r* = −0.789). However, Lin et al ^[21,]^ Salmonowicz et al^[22]^ and Sjogren et al^[38]^ failed to show any relation between duration of diabetes and serum magnesium level in type I diabetic children. This difference may be due to different study populations and variable degree of diabetic control among these populations.

In our study, there was a significant difference in age between hypomagnesemic and normomagnesemic diabetic children, as mean age of diabetics with low magnesium was (4.82 ± 2.35 years) versus (1.94 ± 1.45 years) in those with normal magnesium level (*P* < 0.001). This is in agreement with Mishra et al ^[35]^ where they found a positive correlation between age of T2DM adults and serum magnesium level (*r* = 0.721) (*P* < 0.001) but disagreed with Lin et al ^[21]^ and Salmonowicz et al ^[22]^ who showed no correlation between age of T1DM children and serum magnesium level. This disagreement could be explained by different study populations between their studies and ours. Wang et al ^[34]^ showed similar results to Lin et al ^[21]^ and Salmonowicz et al ^[22]^ but in type 2 diabetes patients. Jiancheng et al ^[20]^ stated that in adults with T2DM; older age is seen more frequent among the normomagnesemic group.

## 5 Conclusion

We conclude that total serum magnesium is frequently low in Egyptian children with type 1 diabetes and it is correlated with glycemic control and with lipid profile. Hypomagnesemia was more evident in patients with poor diabetic control and those with higher atherogenic lipid parameters. We suggest regular monitoring of serum magnesium in children with type 1diabetes and correcting hypomagnesemia if present. We need to perform further studies on giving magnesium supplements in diabetic children with hypomagnesemia to observe the effect of correction of serum magnesium on glycemic control, lipid profile, and the risk of diabetic complications.

### 5.1 Study limitations

Several limitations of this study deserve mentioning. First, we did not measure erythrocyte magnesium content. As magnesium is a predominantly intracellular ion, its serum measurements are not representative of magnesium status or intracellular pool. In this regard, significant intracellular magnesium depletion could be seen with normal serum concentrations^[39]^; however, because we already having a higher percentage of hypomagnesaemia in serum of our diabetic patients compared to our control subjects, this potential limitation did not influence our objective and conclusions. Second we did not randomize our patients, instead, we took all patients coming consecutively to our outpatient Endocrinology unit. However, because we have a good number of patients with age and sex matched control, this potential limitation did not influence our objective and conclusions. We recommend performing further multicenter studies on different populations with different levels of glycemic control in order to get more accurate results regarding the relationship between serum and intracellular magnesium in children with type 1Diabetes.

## References

[1] Atkinson MA Eisenbarth GS Type 1 diabetes: new perspectives on disease pathogenesis and treatment. Lancet 2001;358:221–9. 11476858 10.1016/S0140-6736(01)05415-0

[2] Bluestone JA Herold K Eisenbarth G Genetics, pathogenesis and clinical interventions in type 1 diabetes. Nature 2010;464:1293–300. 20432533 10.1038/nature08933 PMC4959889

[3] Mark A Atkinson The pathogenesis and natural history of type 1 diabetes. Cold Spring Harb Perspect Med 2012;2:a007641. 23125199 10.1101/cshperspect.a007641 PMC3543105

[4] Kiess W Böttner A Raile K Type 2 diabetes mellitus in children and adolescents: a review from a European perspective. Horm Res 2003;59 suppl 1:77–84. 12566725 10.1159/000067829

[5] Florez JC Clinical review: the genetics of type 2 diabetes: a realistic appraisal in 2008. J Clin Endocrinol Metab 2008;93:4633–42. 18782870 10.1210/jc.2008-1345 PMC2626447

[6] Thomas Reinehr Type 2 diabetes mellitus in children and adolescents. World J Diabetes 2013;4:270–81. 24379917 10.4239/wjd.v4.i6.270 PMC3874486

[7] Tapiero H Tew KD Trace elements in human physiology and pathology: zinc and metallothioneins. Biomed Pharmacother 2003;57:399–411. 14652165 10.1016/s0753-3322(03)00081-7

[8] Rasheed H Elahi S Ajaz H Serum magnesium and atherogenic lipid fractions in type II diabetic patients of Lahore, Pakistan. Biol Trace Elem Res 2012;148:165–9. 22351157 10.1007/s12011-012-9361-5

[9] Kaplinsky C Alon US Magnesium homeostasis and hypomagnesemia in children with malignancy. Pediatr Blood Cancer 2013;60:734–40. 23303583 10.1002/pbc.24460

[10] Lippincott Williams and Wilkins, Bishop ML Fody EP Schoef FL Clinical Chemistry, Principles, Procedures and Correlations. 2005;268–269; 327–330.

[11] Rude RK Magnesium deficiency and diabetes mellitus. Causes and effects. Postgrad Med 1992;92:217–24. 10.1080/00325481.1992.11701494 1409173

[12] Dasgupta A Sarma D Saikia UK Hypomagnesemia in type 2 diabetes mellitus. Indian J Endocrinol Metab 2012;16:1000–3. 23226651 10.4103/2230-8210.103020 PMC3510925

[13] Johansson G Danielson BG Ljunghall S Evidence for a disturbed magnesium metabolism in diabetes mellitus. Magnes Bull 1981;2:178–80.

[14] Sjogren A Florén CH Nilsson A Oral administration of magnesium hydroxide to subjects with insulin-dependent diabetes mellitus: effects on magnesium and potassium levels and on insulin requirements. Magnesium 1988;7:117–22. 3054347

[15] Djurhuus MS Henriksen JE Klitgaard NA Effect of moderate improvement in metabolic control on magnesium, lipid concentrations in patients with type 1 diabetes. Diabetes Care 1999;22:546–54. 10189530 10.2337/diacare.22.4.546

[16] Deckert T Feldt-Rasmussen B Borch-Johnsen K Albuminuria reflects widespread vascular damage. The Steno hypothesis. Diabetologia 1989;32:219–26. 2668076 10.1007/BF00285287

[17] Galli-Tsinopoulou A Maggana I Association between magnesium concentration and HbA1c in children and adolescents with type 1 diabetes mellitus. J Diabetes 2014;6:369–77. 24393429 10.1111/1753-0407.12118

[18] Agrawal P Arora S Singh B Association of macrovascular complications of type 2 diabetes mellitus with serum magnesium levels. Diabetes Metab Syndr 2011;5:41–4. 22814841 10.1016/j.dsx.2010.12.003

[19] Suárez A Pulido N Casla A Impaired tyrosine-kinase activity of muscle insulin receptors from hypomagnesaemic rats. Diabetologia 1995;38:1262–70. 8582534 10.1007/BF00401757

[20] Jiancheng Xu Wei Xu Hanxin Yao Associations of serum and urinary magnesium with the pre-diabetes, diabetes and diabetic complications in the Chinese Northeast Population. PLoS One 2013;8:56–7. 10.1371/journal.pone.0056750 PMC3572031 23418599

[21] Lin CC Tsweng GJ Lee CF Magnesium, zinc, chromium levels in children, adolescents, young adults with type 1 diabetes. Clin Nutr 2016;35:880–4. 26096861 10.1016/j.clnu.2015.05.022

[22] Salmonowicz B Krzystek-Korpacka M Noczyńska A Trace elements, magnesium, and the efficacy of antioxidant systems in children with type 1 diabetes mellitus and in their siblings. Adv Clin Exp Med 2014;23:259–68. 24913117 10.17219/acem/37074

[23] Bjelakovic G Sokolovic D Ljiljana S Arginase activity and magnesium levels in the blood of children with diabetes mellitus. J Basic Clin Physiol Pharmacol 2009;20:319–34. 20214019 10.1515/jbcpp.2009.20.4.319

[24] Fort P Lifshitz F Magnesium status in children with insulin dependent diabetes mellitus. J Am Coll Nutr 1986;5:69–78. 3700884 10.1080/07315724.1986.10720114

[25] Tuvemo T Ewald U Kobbah M Serum magnesium and protein concentrations during the first five years of insulin-dependent diabetes in children. Acta Paediatr Suppl 1997;418:7–10. 9055931 10.1111/j.1651-2227.1997.tb18297.x

[26] Seyoum B Siraj ES Saenz C Hypomagnesemia in Ethiopians with diabetes mellitus. Ethn Dis 2008;18:147–51. 18507265

[27] Hussmann MJ Fuchs P Truttmann AC Extracellular magnesium depletion in pediatric patients with insulin-dependent diabetes mellitus. Miner Electrolyte Metab 1997;23:121–4. 9252979

[28] Matthiesen G Olofsson K Rudnicki M Ionized magnesium in Danish children with type 1 diabetes. Diabetes Care 2004;27:1216–7. 15111552 10.2337/diacare.27.5.1216

[29] Mikhail N Ehsanipoor K Ionized serum magnesium in type 2 diabetes mellitus: its correlation with total serum magnesium, hemoglobin A1c levels. South Med J 1999;92:1162–6. 10624906 10.1097/00007611-199912000-00005

[30] Roffi M Kanaka C Mullis PE Bianchetti MG: hypermagnesiuria in children with newly diagnosed insulin-dependent diabetes mellitus. Am J Nephrol 1994;14:201–6. 7977481 10.1159/000168715

[31] Ramadass S Basu S Srinivasan AR Serum magnesium level as an indicator of status of diabetes mellitus type 2 diabetes. Metab Syndr 2015;9:42–5. 10.1016/j.dsx.2014.04.024 25470649

[32] Sinha S Sen S Status of zinc and magnesium levels in type 2 diabetes mellitus and its relationship with glycemic status. Int J Diabetes Developing Countries 2014;34:220–3.

[33] Wegner M Araszkiewicz A Zozulińska-Ziółkiewicz D The relationship between concentrations of magnesium and oxidized low density lipoprotein and the activity of platelet activating factor acetylhydrolase in the serum of patients with type 1 diabetes. Magnes Res 2010;23:97–104. 20507838 10.1684/mrh.2010.0207

[34] Wang S Hou X Liu Y Serum electrolyte levels in relation to macrovascular complications in Chinese patients with diabetes mellitus. Cardiovasc Diabetol 2013;10:146. 10.1186/1475-2840-12-146 PMC3852555 24112518

[35] Mishra S Padmanaban P Deepti GN Serum magnesium, dyslipidemia in type-2 diabetes mellitus. Biomed Res 2012;23:295–300.

[36] Srinivasan AR Niranjan G Kuzhandai Velu V Status of serum magnesium in type 2 diabetes mellitus with particular reference to serum triacylglycerol levels. Diabetes Metab Syndr 2012;6:187–9. 23199535 10.1016/j.dsx.2012.09.001

[37] Guerrero-Romero F Rodriguez-Moran M Hypomagnesemia is linked to low serum HDL-cholesterol irrespective of serum glucose values. J Diabetes Complications 2000;14:272–6. 11113690 10.1016/s1056-8727(00)00127-6

[38] Sjögren A Florén CH Nilsson A Magnesium deficiency in IDDM related to level of glycosylated hemoglobin. Diabetes 1986;35:459–63. 3956882 10.2337/diab.35.4.459

[39] Reinhart R Marx J Haas R Intracellular magnesium of mononuclear cells from venous blood of clinical healthy subjects. Clin Chim Acta 1988;48:2415–20. 10.1016/0009-8981(87)90371-8 3665095

[40] Rewers M Pihoker C Dohaghue K Assessment and monitoring of glycemic control in children and adolescents with diabetes. Pediatr Diabetes 2009;10:71–81. 19754620 10.1111/j.1399-5448.2009.00582.x

